# Farmers and Local Residents Collaborate: Application of a Participatory Citizen Science Approach to Characterising Air Quality in a Rural Area in The Netherlands

**DOI:** 10.3390/s22208053

**Published:** 2022-10-21

**Authors:** Amber Woutersen, Henri de Ruiter, Joost Wesseling, Wouter Hendricx, Christa Blokhuis, Sjoerd van Ratingen, Kirsten Vegt, Marita Voogt

**Affiliations:** 1 National Institute for Public Health and the Environment (RIVM), P.O. Box 1, 3720 BA Bilthoven, The Netherlands; 2 Consumption and Healthy Lifestyles, Department of Social Sciences, Wageningen University & Research, P.O. Box 8130, 6700 EW Wageningen, The Netherlands

**Keywords:** citizen science, air quality, citizen sensing, agriculture, PM, NO_2_, NH_3_, sensors, Palmes, stakeholders, odour annoyance

## Abstract

In rural areas, livestock farming is a source of environmental concern. We describe a citizen science (CS) project in Venray, the Netherlands, where air quality was measured at livestock farms and surrounding residential premises. We used low-cost methods to measure air quality components and facilitated a dialogue between stakeholders about the results and solutions for cleaner air. PM_2.5_ and PM_10_ were measured using Nova Fitness SDS011 sensors, nitrogen dioxide (NO_2_) and ammonia (NH_3_) using Palmes tubes and odour annoyance was reported. Particulate Matter (PM) concentrations were higher close to layer farms, but elevated concentrations were limited at other farms and residential locations. NO_2_ concentrations were elevated near busy roads, and higher NH_3_ values were measured near livestock farms. Reporting of odour annoyance was limited, yet during the dialogue residents indicated that this was their largest concern. While both farmers and residents agreed with the general conclusions, they still preferred opposing measures. We conclude that characterisation of air quality using low-cost methods is possible, but expert guidance is needed. Moreover, education, commitment of participants and involvement of independent parties are crucial to ensuring a productive dialogue between stakeholders. The insights gained by participants and resulting dialogue were the greatest benefits of this CS approach.

## 1. Introduction

In rural/agricultural areas, livestock farming is a source of environmental concern [1]. Among residents exposure to air pollution has raised health concerns, and exposure to odour has led to nuisance (odour annoyance; [2,3,4]). This may in turn cause tension between residents and farmers [2,3]. Air polluting substances have previously been measured inside livestock stables in order to establish emission factors (e.g., [5,6]). However, measurements of air quality and odour in the surroundings of livestock farms are sparce. We found one study that performed low time resolution (two week period) PM_10_ measurements at 61 residential sites in livestock-dense areas by collecting dust on filters [7]. They found limited variation in PM_10_ concentrations between residential locations, indicating small spatial variation. Besides air pollution, odour annoyance is often a problem in rural areas. It is still difficult to measure with low-cost, easy-to-use sensors, which is why the evaluation of citizens’ exposure to odour has increasingly been based on modelling approaches [8]. There have been attempts to develop sensors that measure (combinations of) scented substances from livestock farming, and these sensors have been the subject of research for many years focussing on application inside livestock stables (e.g., [9,10]). However, this has not yet resulted in commercially available sensors, which makes the high temporal resolution measurement of odours at residential locations difficult.

In the Netherlands, air quality is assessed with the aid of models that are calibrated using measurements from the national air quality monitoring network [11]. There are few measurement locations in rural areas. In 2018, inhabitants of the Municipality of Venray, situated in an area with intensive livestock farming operations in the southeast of the Netherlands, expressed the need for more measurements in their region since they experience odour annoyance due to livestock farming and worry about the air quality. They contacted the National Institute of Public Health and Environment (Dutch: Rijksinstituut voor Volksgezondheid en Milieu; RIVM), which is responsible for the national air quality monitoring network, for advice on how to monitor air quality in their living environment. Around the same time as the inhabitants of Venray contacted RIVM, the Limburg Agricultural and Horticultural Association (Dutch: ‘Limburgse Land- en Tuinbouwbond’; LLTB; the regional organisation representing the interests of farmers) asked RIVM to participate in a pilot study on the application of low-cost sensors. This pilot study was aimed at gaining greater insight into the contribution of local sources to air pollution levels. Under the applicable legal frameworks, the emissions from livestock stables (e.g., of PM and NH_3_) must be calculated based on standardised emission factors [12], but according to LLTB, farmers want to be held accountable for their actual, measurable emissions. Additionally, farmers want to know what other sources than farming contribute to air pollution, since they believe they are not the only polluters. A situation arose in which both farmers and local inhabitants wanted air quality measurements to be performed in their area. This offered a unique opportunity to perform a CS study in which stakeholders with different needs and interests participated, including the party that was viewed as the polluter by the residents. 

In recent years, the number of environmental CS initiatives in which citizens use sensors to measure pollution levels in their environment has increased significantly [13,14,15,16]. Simultaneously, the market for measuring devices has developed significantly, and there are now multiple sensors available that are both affordable and easy to use. People have multiple reasons to measure pollution levels in their environment: they may be concerned about the impact of the environment on their health, distrust official governmental measurements, or are interested in the technical aspects of performing measurements [15]. These incentives are in keeping with the characteristics of *citizen sensing*, which falls under the broader umbrella of citizen science. Suman (2020; [17]) defined citizen sensing as “a form of grassroots-driven monitoring initiatives aimed at tracking environmental factors (in alternative or in addition to official governmental monitoring), making use of Information and Communication Technology (ICT), in general, and, in particular, of sensors.”

For air quality specifically, there are several affordable sensors available, most of which measure PM using optical particle counters. PM stems from many sources, including livestock farming and wood burning, which tend to exist in rural areas. Examples of PM sensors are the SDS011 (Nova Fitness Co., Jinan, Shandong Province, China) and the SPS30 (Sensirion AG, Zurich, Switzerland). These devices are cheap (±€10–50), readily available and easy to use. However, they are known to overestimate concentrations under conditions of high relative humidity (RH) [18,19,20], and their ability to detect larger particles (>2.5 µm) varies [21]. Calibration algorithms can be used to compensate for these shortcomings, but can only do so in part [20]. Other air pollutants that occur in rural areas are NO_2_ and NH_3_. NO_2_ is a good indicator of road traffic as a source of air pollution and NH_3_ of (livestock) farming. There are a few real-time electrochemical sensors on the market that can detect NO_2_ concentrations (e.g., NO2-A43F, Alphasense, UK). However, this type of sensor exhibits cross sensitivity to other gases like ozone, and temperature and humidity [18], making its application in a CS context challenging. An alternative to real-time NO_2_ sensors is the well-known low-cost method that uses diffusion tubes (e.g., [22]), whereby an average NO_2_ concentration is generated for the period during which the tubes are exposed. The same holds for measuring NH_3_; real-time sensors for ambient air are still in the development phase, but the use of Palmes tubes is a common method for gathering data for periodic average concentrations (e.g., [20]). 

There is no standard format for CS projects, and initiatives may therefore exist in many different forms [14]. Here, we developed our own approach with the following objectives: (a) to address the concerns of both farmers and residents; (b) to actively involve both farmers and residents and facilitate a dialogue about possible solutions for improving the local air quality and reducing odour annoyance; (c) to rely on low-cost methods to enable multiple participants to be involved and perform measurements on their premises; and (d) to measure multiple components simultaneously in order to characterise air quality and odour annoyance in relation to different sources of air pollution. 

The objective of this paper is (1) to present the results of the air quality measurements, (2) to communicate the lessons learned about the low-cost measurement methods and their applicability in this CS project, and (3) to share our observations about the insights gained by the dialogue between farmers and residents throughout the project.

## 2. Materials and Methods

### 2.1. Project Design

After the first inquiry in 2018, the project ‘Farmers and Neighbours’ (Dutch: ‘Boeren en Buren’) started in 2019. The entire project lasted for 2.5 years, of which one year was spent carrying out the measurements. This project was carried out by RIVM in cooperation with LLTB (representing local farmers), Healthy Living Environment Venray (Dutch: ‘Gezond Leefmilieu Venray’; GLV; representing local residents) and the municipality of Venray. The farmer and resident participants were recruited by interest groups LLTB and GLV. Funding for the project was provided by the Strategic Programme RIVM and the municipality of Venray partly paid for the material costs. RIVM was in charge of project management, provided data management and data analyses, formulated the conclusions about the air quality measurements and organised all meetings.

**Figure 1:**
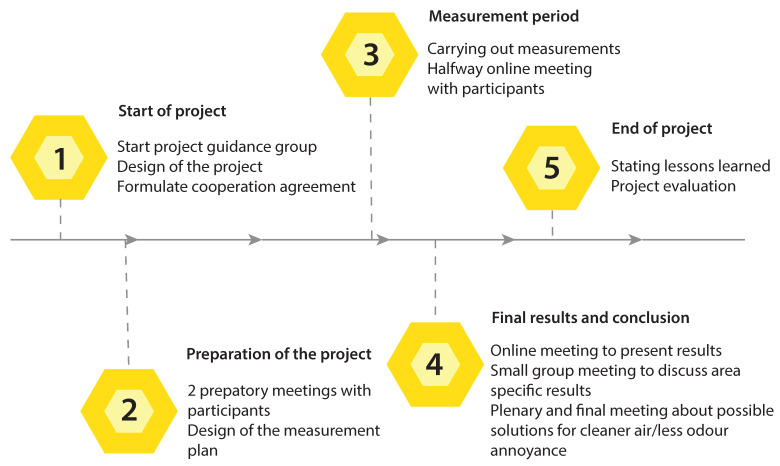
Schematic representation of the different phases of the project and corresponding activities.

We distinguished five different phases during this project: (1) the initiation phase, (2) the preparation phase, (3) the execution phase, (4) the concluding phase, and (5) the evaluation phase (Figure 1). In the **first phase**, representatives of RIVM, LLTB, GLV and the municipality of Venray started a project guidance group and discussed how to design the project. All parties agreed that the implementation of results and possible solutions was explicitly not part of this project, since this depends on local, regional and even national political readiness. Project partners repeated this message throughout the project. In this first phase the guidance group also compiled a detailed cooperation agreement setting out joint interests, party-specific interests, project goals and specific agreements for the execution of the project. We used this document throughout the project to reflect on the initial agreements and for resolving conflicting situations. In the **second phase**, we organised two preparatory meetings with the participants (inhabitants and farmers) in the city hall of Venray, during which interaction between the participants and the experts was facilitated using group conversations and digital interaction services (e.g., Mentimeter; ©Mentimeter, Stockholm, Sweden). The participants were asked what questions they had that could be answered by the measurements. Experts from RIVM used this input to develop the measurement plan. Five participants volunteered as contact persons for each of the study areas and to replace the Palmes tubes in their area. One participant volunteered to offer technical support to participants that experienced technical defects or difficulties with their sensor. In the **third phase**, the measurements were carried out. This period lasted one year, to ensure that all seasons and corresponding weather conditions, as well as seasonal sources of air pollution were included. In this phase we organised one meeting with the participants to discuss the interim measurement results, after 6 months of measuring. Due to COVID-19 restrictions, we were forced to organise this meeting online. In the **fourth phase**, the final results and conclusions drawn by RIVM were discussed with the participants. This was again done online, due to COVID-19 restrictions. Eventually, nine months after the measurements were finished, the national government eased the COVID-19 restrictions and RIVM organised a physical meeting in the city hall of Venray, during which small groups discussed the results and began discussing solutions that would result in cleaner air and reduce odour annoyance. The dialogue was continued during a plenary and final meeting in October 2021. During the **fifth phase**, the lessons learned were set out and the project was evaluated. Participants were not included in this phase, but they were represented by their representatives from project guidance group. During all phases, representatives of each participating party met on a regular basis for a project guidance group meeting. See Supplementary Table S1 for a complete overview of all of the meetings that took place during the project. 

### 2.2. Study Area and Measurement Locations

**Figure 2:**
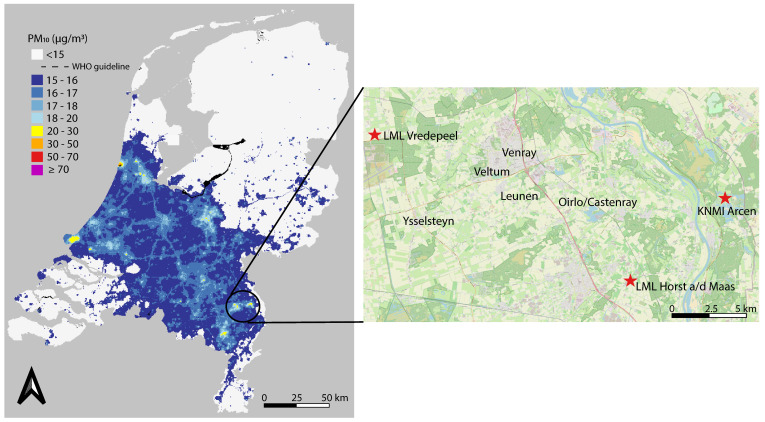
Map with average PM concentrations (µm/m^3^) for the year 2019 and a map of Venray and its surroundings (area indicated on the PM map by the circle). The five areas where measurements were carried out are indicated on the map on the right, as well as the two official measurement stations and the KNMI weather station (red stars). ©OpenStreetMap contributors. Source of PM map: https://www.atlasleefomgeving.nl/kaarten (accessed on 16 March 2022).

The study was carried out in Venray, an urban municipality surrounded by thirteen rural villages, which have a total of approximately 44,000 inhabitants. The soil in the area is sandy and the region has relatively high PM concentrations compared to other parts of the Netherlands (Figure 2). Furthermore, there is a high density of livestock farms, of which a relatively large number are poultry (layers and broilers) and pig farms. Livestock farms are therefore expected to be sources of air pollution, but domestic wood burning, the highway and provincial roads are expected to contribute as well. Venray does not have any large industrial sites. Measurements for this study were carried out in the towns Venray (city), Veltum, Ysselsteyn, Oirlo/Castenray and Leunen, resulting in a study area measuring approximately 12 × 11.5 km (Figure 2). In total, 4 livestock farmers (2 with layers, 1 with broilers and 1 with pigs) and 26 residents participated. This resulted in 33 locations with PM sensors, 18 locations with NO_2_ Palmes tubes and 37 locations with NH_3_ Palmes tubes, divided over the different areas. The participants chose the measurement locations themselves, preconditioned by possibilities for power supply, following some guidelines provided by RIVM based on previous experience (e.g., avoid full direct sunlight or humid locations where possible). The participating farmer in Leunen placed three PM sensors in close proximity (35–100 m) to the exhaust fan of his layer stable, in order to investigate the decrease in PM concentration with distance to the exhaust fan. Exhaust fans are installed to ventilate stables, in order to keep the environment healthy for the livestock. Consequently, particles and gases (e.g., NH_3_) from within the stables are transported to the ambient air through these fans. Depending on the stable, the air is treated to decrease the amount of particles and gases just before the air is emitted to the ambient air. One of these sensors close to the fan was disconnected from the power supply for long periods of time and was therefore discarded. The three other farmers placed their two PM sensors at 100–150 m from the exhaust fan or side outlet of their stables. One of these sensors was discarded because it had no power supply for a long period of time. 

### 2.3. Particulate Matter

**Figure 3:**
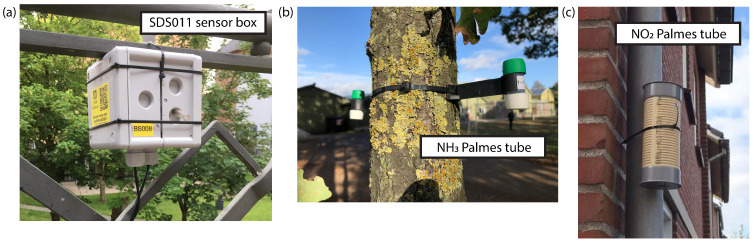
Photographs of PM sensorbox and Palmes tubes. (**a**) SDS011 sensorbox attached to a balustrade; (**b**) NH_3_ Palmes tubes attached to a tree; (**c**) NO_2_ Palmes tube, placed in a small shelter to minimize the influence of (traffic induced) turbulence.

All measurements were carried out from November 2019 to October 2020. PM (PM_2.5_ and PM_10_) was measured with an SDS011 sensor (Nova Fitness Co., CN), housed in a Kopp electrical junction box with short inlet tubes made of non-conductive materials (Figure 3a). The sensor was connected to a SARA AFF N211 microcontroller containing a NB-IoT communication module manufactured by SODAQ. The readings were averaged every 12 min and transmitted via NB-IoT. The data were aggregated into hourly values and saved in a database in which RIVM collects sensor measurements performed in the Netherlands. We used the SDS011 type because it is the most commonly used sensor in the Netherlands and a nationwide on-the-fly hourly calibration algorithm was already available for this type at the start of the project. RIVM has developed this algorithm in order to deal with the susceptibility of the SDS011 sensor to humidity (“method 2” in Wesseling et al., 2019 [20]). For the calibration, at each official monitoring station in The Netherlands SDS011 sensors situated in an area several kilometres from the station are selected. The average concentration of these sensors is then compared to the official measured concentration and the ratio between them is calculated. The ratio values are interpolated between the locations of the official monitoring station in order to get the calibration factor at the sensor location of interest. The measurements in this study were calibrated accordingly. Statistical analyses of the calibration of the SDS011 sensor data were presented by Wesseling et al. (2021 [23,24]). They checked the calibration procedure by comparing the calibrated SDS011 data collected in the Netherlands in 2020 with data from the nearest station of the national air quality monitoring network, while disregarding data from the nearest station in the calibration procedure. For most sensors in the Netherlands, relatively high correlation coefficients and small biases indicated considerable agreement between the calibrated SDS011 and official data. However, some individual sensors showed a low correlation and typically sensors located in our study area, close to official station NL10131 “Vredepeel”, overestimated concentrations when this station was left out of the calibration. 

In our study, we applied the calibration algorithm that included the nearby official station (“Vredepeel”; Figure 2). The other nearby station (“Horst aan de Maas”; Figure 2) was not included in the calibration algorithm due to the absence of sensors in its vicinity. When comparing the resulting values of the sensors with official measurements at the two official stations nearby, we found that the sensors still substantially overestimated the values after calibration under conditions of very high relative humidity (Supplementary Information S1). We therefore only used PM measurements when relative humidity was equal to or below 95%, as measured at nearby KNMI weather station Arcen (KNMI station 391; Figure 2). In this way, we were able to strike a balance between limiting the number of hours for which measurements were disregarded and leaving out measurements taken during the hours in which the overestimation was most pronounced. 

Since low-cost PM sensors often respond erratically to either high relative humidity, disturbances in the power connection, or dust collection in the inlet/outlet, we used duplicate sensors for each location, allowing us to validate concentrations and analyse the uncertainty (see Supplementary Information S2 for method and results of this analysis). During the measurement period, malfunctioning sensors were visited by the appointed participant to service them or replace them, following instructions by RIVM experts. After calibration, duplicate measurements of sensor pairs were compared and measurements from single sensors were rejected when the difference between duplicates was greater than 100 µg/m^3^ (both for PM_2.5_ and PM_10_) in order to filter out the clearly erroneous measurements. Subsequently, time series of neighbouring (duplicate and single) sensors were visually intercompared. The rejection criteria for removal were (1) short-term peaks from single sensors that, in the absence of an operational duplicate sensor at residential locations, were not measured by the neighbouring sensors and (2) long-term deviations between the duplicate sensors greater than 40%. Finally, validated duplicate values were averaged.

All PM data were available as real-time open data on Samen Meten—Dataportaal (sensors.rivm.nl (accessed on 20 April 2022)), except the data from the two sensors that were within 100 metres of the stable exhaust fan in Leunen (after reaching specific agreements with the farmer). 

### 2.4. Nitrogen Dioxide and Ammonia

NO_2_ and NH_3_ were measured on a monthly basis using Palmes tubes (Figure 3b,c; [22]). Unlike the PM sensors, the Palmes tube method is well-known, widely used for monitoring purposes, and RIVM also uses these types of measurements in the National Air Quality Monitoring Network [25] and the Measuring Ammonia in Nature (MAN) network [26,27]. This means that these measurements provide a better indication of absolute values than the PM measurements.

Tubes were collected and put in place by the designated participants. NO_2_ samples were sent to Buro Blauw (the Netherlands) and NH_3_ samples to Gradko International (UK) for analysis. RIVM calibrated the data using the factors determined based on the comparison of Palmes tube measurements at official monitoring stations in the Netherlands with the official data recorded at these stations on a monthly basis. The 95% Confidence Interval (CI) uncertainty in the calibrated yearly average is in the order of 20–25% [25,27]. Calibration might not work as well for values of NH_3_ over 20 µg/m^3^ because the calibration is based on the values recorded at official stations, which are generally less than 20 µg/m^3^. Nevertheless, after calibration the tubes provide reliable indications of the concentration [28].

Data were rejected if the tube was incorrectly installed or returned. Furthermore, in line with the methods of Lolkema et al. (2015; [26]), we rejected monthly values that deviated by over 3 times the standard deviations (3sd) from the 12-month average at the specific location if the deviating value could not be explained by local conditions. 

All NO_2_ and NH_3_ data were available as open data on sensors.rivm.nl on a monthly basis.

### 2.5. Odour Annoyance

We developed a smart-phone app for participants to report odour annoyance. When reporting the odour, the participants were asked how (un)pleasant they found the smell on a 9-interval scale (−4 to +4). These data were saved, with a time-stamp and location. Data were only accessible to RIVM, in order to ensure the independence of the reports. We analysed the possible presence of local sources by constructing wind rose diagrams in which the number of reports were plotted against the occurring wind direction at the moment of the report. Normalising the results for the wind direction frequency distribution enabled us to perform a more representative analysis, since it gives more weight to reports that were made during wind directions that occurred less often.

### 2.6. Data Analysis

Data analysis and interpretation were performed by experts from RIVM. In addition, participants were encouraged to analyse the data themselves. For this purpose, the RIVM Shiny App ‘Samen Analyseren Tool’ (analyseren.samenmeten.nl), based on the R package openair [29], was used for the PM sensor data and made available to the participants. This tool is aimed at presenting complex air quality data in a way that is easy for non-experts to understand. The tool made it relatively easy for participants to generate relevant figures, such as time series and pollution roses of selected (groups of) sensors. The tool also included data about the wind direction, which made it possible to identify local sources of air pollution. We used meteorological observations from the official KNMI weather station in Arcen (Figure 2), situated around 10 km to the east of the study area. The wind data were also used in the analysis of the odour reports using the polar plot function from the ggplot2 library in Rstudio [30]. During the online webinars and live meetings in which we discussed the measurement results, RIVM also used figures that were generated with the tool, and were thus available to all participants. The tool is open source and available on https://github.com/rivm-syso/Analyse-Together (accessed on 20 April 2022). Analysis of NO_2_ and NH_3_ measurements was done in Microsoft Excel. The documents were provided to the participants, as well. Furthermore, maps were created using QGIS version 3.16.4.

## 3. Results and Discussion

### 3.1. Measured Air Quality and Odour Annoyance

#### 3.1.1. PM Concentration

**Figure 4:**
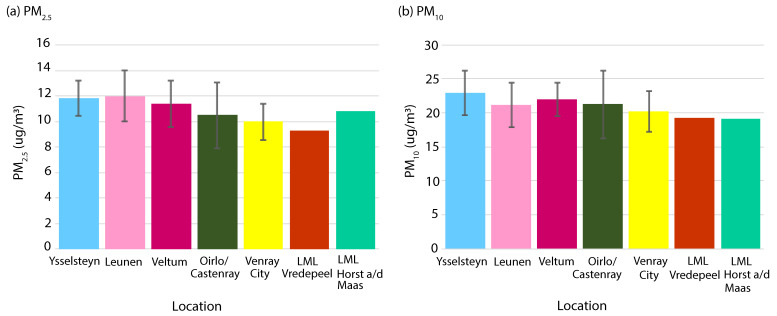
Yearly average concentrations of PM_2.5_ and PM_10_. Average concentrations for the five areas and for the two nearby official measurement stations (Vredepeel and Horst aan de Maas). 2 times standard deviation (95% CI; shown by the error bars) is included.

Yearly values of PM_2.5_ and PM_10_ at residential locations, averaged per area, were in the order of 10–12 µg/m^3^ and 20–23 µg/m^3^, respectively (Figure 4). The figure shows that spatial differences were relatively small. They are in the same order of magnitude as the measurement uncertainty (quantified as the 95%CI of the yearly averaged concentration difference between two collocated sensors at 34 locations) of 4.4 µg/m^3^ for PM_2.5_ and 6.8 µg/m^3^ for PM_10_ (Supplementary Information S2) and therefore not statistically significant. We measured a PM_10_ concentration of 31 µg/m^3^ at 35 m from the exhaust fan of the layer farm in Leunen (not shown). At all other farm locations (at distances larger than 100 m from exhaust locations), yearly average concentrations were not elevated compared to the concentrations at residential locations. 

Using the higher temporal resolution results, the PM sensors prove their value in gaining more insight into the impact of local sources on concentrations, as compared to yearly average concentrations. Based on the hourly values, pollution roses and time series provided detailed insight into the variation in local concentrations as a result of livestock farm emissions. The sensors close to the chicken broiler farm and to the pig farm did not record any increased PM concentrations compared to the surrounding sensors. By contrast, the sensors close to the two layer stables measured elevated concentrations when the wind was coming from the direction of the exhaust fan or side outlet of the stable. 

**Figure 5:**
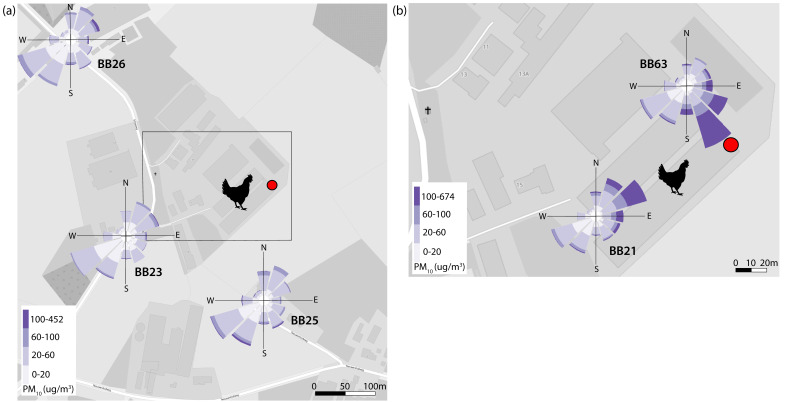
PM_10_ pollution roses recorded by sensors in Leunen at (**a**) residential locations and (**b**) on the farmer’s premises. The average concentration of PM per wind sector is shown by the roses, weighted according to how often wind came from this direction during the period analysed. The locations of chicken stables are indicated by the chicken symbol, and the location of the stable exhaust fan is indicated by the red dot. ©OpenStreetMap contributors.

**Figure 6:**
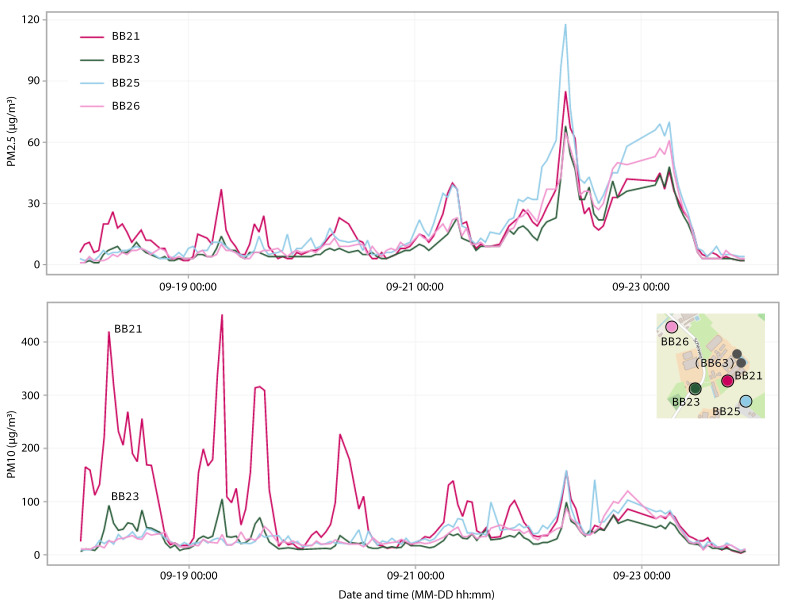
Time series showing PM_2.5_ and PM_10_ concentrations over the course of four days for four sensors in Leunen. No measurements were taken by sensor BB63 during this period.

We present data from the PM_10_ pollution roses (Figure 5) and PM time series (Figure 6) registered by sensors located in Leunen. Since the PM sensors at this farm were at the closest distance from the stable, the elevation in PM concentration was most pronounced. In the pollution roses, the two sensors close to the exhaust fan of the laying hen stable recorded elevated concentrations when the wind was coming from the direction of the exhaust fan (Figure 5b). On the other hand, the sensors at the surrounding residential locations did not measure increased concentrations when the wind was coming from the direction of the stable: the pattern for all three resident locations was very similar (Figure 5a). The time series in Figure 6 cover a period of several days in which the wind was coming from the northeast, i.e., from the stable exhaust fan towards the sensors BB21 and BB23. Increased concentrations can be observed in these sensor readings for PM_10_ and to a lesser extent for PM_2.5_. PM emissions from stables are known to contain a relatively high concentration of particles larger than 2.5 µm [6]. The diurnal pattern of the chicken activity is clearly visible: PM_10_ concentrations were not elevated at night when the chickens were inactive but increased during the day when the chickens were active and resuspended particles. The elevation of the PM_10_ concentration was highest closest to the exhaust fan (BB21 at 100 m) and dropped considerably at a distance from the source. At the residential location BB23 (at 250 m) we observed only a small increase compared to location BB21. The timeseries of PM_2.5_ show elevated concentrations during the final days of the selected period. It can be seen that the sensors at all locations measure higher concentrations during these days. This indicates an increase in the regional background concentration rather than the impact of a local source. The difference in concentration between the individual locations during the days with increased PM_2.5_ concentrations reflect the rather high between sampler uncertainty. 

#### 3.1.2. NO_2_ and NH_3_ Concentrations

**Figure 7:**
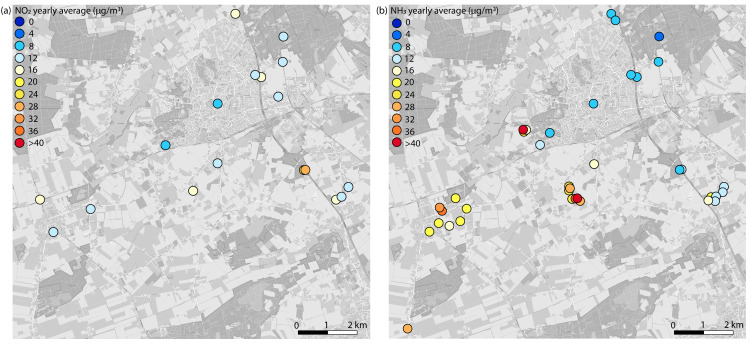
Maps with yearly average concentrations of (**a**) NO_2_ in µg/m^3^ and (**b**) NH_3_ in µg/m^3^. ©OpenStreetMap contributors.

**Figure 8:**
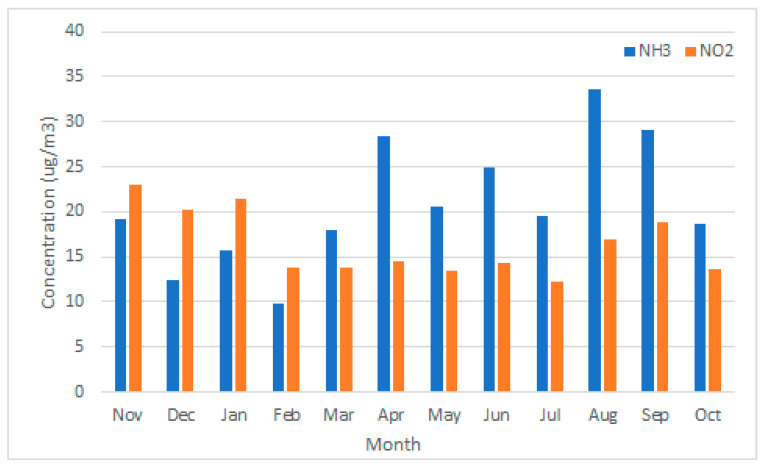
Monthly concentrations of NO_2_ and NH_3_ Palmes measurements. NH_3_ concentrations are shown in blue, NO_2_ concentrations in orange.

The NO_2_ concentration from October 2019 to November 2020 varied between 12–28 µg/m^3^ (Figure 7). The highest concentrations were recorded near the highway and provincial roads. The concentrations of NO_2_ recorded at locations near the highway in the more urban environment of Venray were lower than at the locations near the highway in the rural area to the south of the study area. This may be due to the shielding effect of a noise barrier (as reported in [31,32]). Concentrations were generally higher during the winter months (Figure 8), which can be explained by higher emissions (e.g., from heating) in combination with less dispersion due to more stable atmospheric conditions. 

The NH_3_ concentration from October 2019 to November 2020 varied between 7–58 µg/m^3^ (Figure 7). Two outlying measurements were removed (BB1-9 March and BB3-4 October; both deviated by more than 3sd; see methods). The concentrations recorded in nature reserves and rural areas with a high density of livestock farms differed greatly from each other. At the premises of the farmers, we measured the highest concentrations (up to 58 µg/m^3^). The highway and other principal roads, as well as a sewage treatment plant in the north, proved to be a minor source of NH_3_. The season clearly had an impact on monthly NH_3_ concentrations, as is shown in Figure 8. For example, fertiliser is distributed across fields from March to September, which were also the months in which the highest NH_3_ concentrations were observed.

#### 3.1.3. Odour Reports

**Figure 9:**
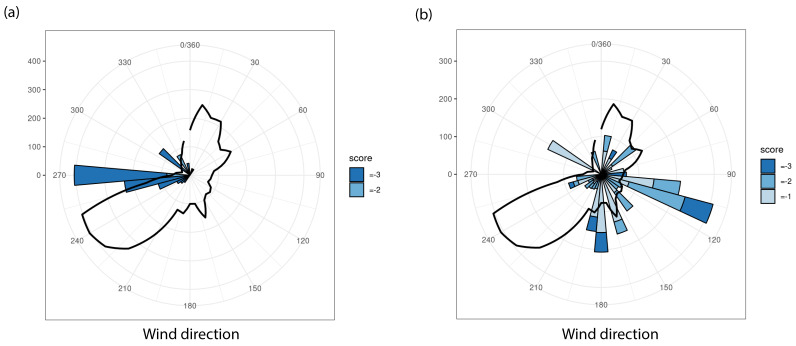
Examples of wind rose diagrams presenting the number of odour annoyance reports per wind sector, normalised for the frequency distribution. The black line represents the wind direction frequency distribution. The score (−1, −2 and −3) indicates the degree of unpleasantness of the odour experienced by the person who made the report (the more negative the value, the more unpleasant the odour).

During the six-month period in which the odour smartphone app was available, 292 reports were made. Only three participants were responsible for 80% of the reports. This bias in reporting and the fact that there was not enough data meant that it was not possible to study odour annoyance comprehensively. Still, we deduced some interesting local insights from the results. Figure 9a is an example of a situation in one of the measurement areas in which nearly all reports were made when the wind came from a westerly direction. To the west of the reporting participant, there was an isolated livestock stable. In this case, the stable could be identified as a local odour source. Figure 9b is an example of a situation in another measurement area in which the reports were made with multiple wind directions. This indicates multiple sources which complicated the identification of the source(s) that caused the odour annoyance.

### 3.2. Lessons Learned about the Low-Cost Methods for Characterising Local Air Quality and Their Applicability in a CS Setting

During the CS project we gained new insights into the different low-cost methods, and learned from the application in a CS project. This study showed that ensuring the proper operation of the PM sensors and obtaining data suitable for analysis required a significant amount of effort. During the one-year measuring period, about 20 sensors malfunctioned as a result of dust collection in the inlet or internal system. The participant who volunteered to deal with malfunctioning sensors spent considerable time on maintenance (both cleaning and replacing defective sensors and cleaning all inlet tubes halfway through the measurement period). Due to the high sensitivity of the PM sensors to humid conditions, we found that applying the on-the-fly generic calibration algorithm [20] was not sufficient in our study area, in contrast to other regions in the Netherlands [23]. It was necessary to apply an RH threshold value, after which the hours used in the data analysis were selected. We have not yet been able to find an explanation for this, and future research may clarify this problem. The project proved that the maintenance of the sensors can be done by non-experts. However, expert knowledge is necessary for data validation and calibration. The need for expert involvement was also demonstrated in the VAQUUMS project [33], aimed at guidance with regard to the available sensors and their performance, deployments and interpretation. This expert involvement can be costly, and low-cost methods therefore sometimes turn out to be more expensive than expected. It is important to keep this in mind when starting a CS project.

We found that the SDS011 PM sensors were able to detect at least a portion of the particles larger than 2.5 µm (“coarse” PM). This can be deduced from the elevated levels in the PM_10_ signal compared to the PM_2.5_ signal registered by the sensors close to the layer stables. In this study, we used locations of the official monitoring network for the calibration of the PM sensor results [20]. These locations were not as close to sources of coarse PM as the sensors near the stables and were therefore not exposed to the same concentrations of coarse particles. In practice, this means that the PM_10_ values measured by the sensors are likely underestimated, despite the applied calibration algorithm. In a laboratory study the detection efficiency of particles larger than 2.5 µm for the SDS011 was found to be less than 20% [21], supporting the underestimation of PM_10_ values. Performing official PM_10_ measurements in close proximity to coarse PM sources may enhance the calibration, but this needs further investigation. Although absolute PM_10_ values are less accurate, our study showed that the SDS011 sensors are able to detect sources known to emit particles larger than 2.5 µm such as livestock stables, soil blown dust and construction works. This is not the case for multiple other types of sensors. In the field tests performed in the VAQUUMS project, the SDS011 sensor was among the only two types that sometimes detected particles larger than 2.5 µm [34].

It was possible to identify the layer stables as local sources, since the high temporal resolution of the PM sensors enabled the analysis of variations in PM concentrations depending upon the wind direction. However, it was not possible to identify individual smaller sources like other livestock stables, residential wood burning or road traffic using the hourly values. Compared to the PM background levels, the contribution of these smaller local sources turned out to be too limited to be detected at the locations chosen by the participants using PM sensors. It is generally known that in the Netherlands, differences in PM mass concentrations within a region are relatively small compared to the regional background concentration, except at locations close to large industrial or agricultural sources (see Figure 2). For example, Boogaard et al. [35] found a small difference (ratio 1.2) between PM mass concentrations on moderately-busy streets and at urban background locations in the Netherlands, which was in line with the results of earlier studies (e.g., [36,37]). In this respect, the relatively large degree of uncertainty in absolute levels of individual sensors is a disadvantage.

We could not identify local NO_2_ or NH_3_ sources using the same methods as for PM. While the temporal resolution is an advantage of the PM sensors, it is one of the weaknesses when it comes to the Palmes tubes measurements of NO_2_ and NH_3_. Their low temporal resolution (once a month in this study) hampers identification of local, individual sources using the wind direction, since this varies over a one-month period. However, the measurements did make it possible to gain more insight into the contribution of road traffic and livestock stables since their emissions make a relatively larger contribution to the concentrations of NO_2_ and NH_3_, respectively, than to PM levels. Even though all low-cost methods have their own shortcomings, when combined they seem to provide a coherent picture of the overall air quality and sources in the municipality of Venray.

Regarding documenting odour annoyance, we found that the odour reporting smartphone app was not a suitable means for obtaining representative data on moments of odour annoyance during the measurement period. Too few participants consistently made reports throughout the project. Reasons for not using the app were both technical and behavioural in nature. Technical issues related to the app malfunctioning, especially in the initial phase. This prevented several participants from using the app at the beginning, and caused some of them to decide not to use it in later stages, even though most technical issues had been resolved. The more behavioural issue was that participants found it demanding to keep reporting odour annoyance for the duration of the project. They stated that they did not want to consciously spend time on odour annoyance, since that would only increase the stress and discomfort they experienced. Therefore, new methods for measuring or documenting odour annoyance should focus on minimising the effort required of the user. 

### 3.3. Dialogue between Farmers and Residents

An important part of this project was the discussion of the measurement results with the participants, and the new insights this gave them. In order to make this discussion interactive and more insightful for the participants, we printed A0 maps of the villages and provided the participants with transparent A5 graphs (e.g., pollution roses in Figure 5 and odour roses in Figure 9). They could place the transparent graphs of their own sensor on the map and once all graphs were positioned, a conversation was held about the contribution of different sources and the influence of, for example, the wind direction. This was followed by a dialogue between the farmers and residents about possible ways to make the air cleaner.

**Figure 10:**
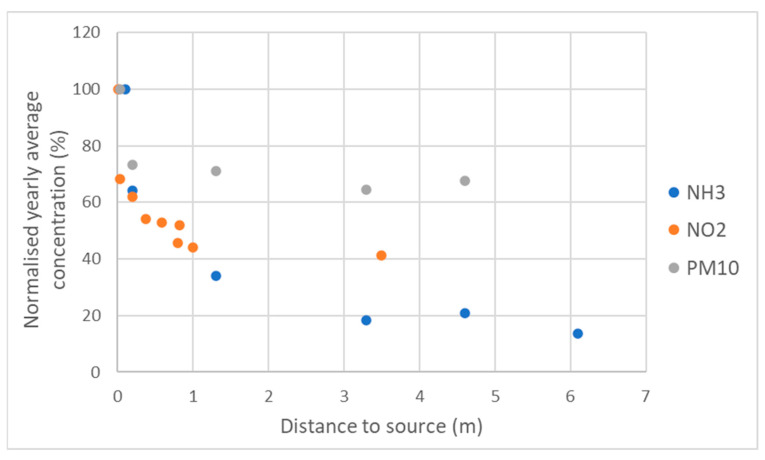
Relative decrease in concentration with increasing distance from the source based on a selection of representative locations of stables (PM_10_ and NH_3_) and traffic (NO_2_). The concentration at distance 0 m is set to 100%.

We found that discussing the measurement results helped participants gain insight into the air quality and pollution sources. For example, although the PM levels measured exceeded the WHO guidelines, some participants did not expect these values to be well below the European legal limits of 20 µg/m^3^ for PM_2.5_ and 40 µg/m^3^ for PM_10_ [38]. They assumed that values exceeding this threshold would be much more common than they actually are. Furthermore, participants discovered that PM generally varies more over time than in space and that the contribution to PM by local sources is relatively low, and higher by NO_2_ and NH_3_ (Figure 10). This insight was also useful in the dialogue about possible solutions. It made the participants realise that in order to decrease PM levels, measures need to be taken on a large scale, in contrast to decreasing NH_3_ emissions, for which eliminating one local source can already lead to a large, local reduction in NH_3_ concentrations. The discussion of the emissions of different substances by different sources was also useful for making participants realise they can also decrease their own emissions (e.g., by not burning wood). 

We feel that the dialogue in this project about possible solutions went well. Participants expressed their different opinions and interests, but the neutral meeting facilitator helped prevent discussions from escalating when things became tense. However, outside the meetings, we observed more tensions between residents and farmers. An upcoming paper on mutual understanding and trust between farmers, local residents and the local government will further investigate how all of the involved parties felt about the dialogue.

During the dialogue, participants used their new insights about air quality in discussing possible ways to improve the air quality. For example, one participant mentioned that she was willing to decrease her domestic wood burning. However, when asked to indicate their preferences for different types of measures, participants generally preferred those that did not require them to take any direct action. For example, they preferred measures that would require governments to reduce the use of fossil fuels for energy generation to measures that would require them to reduce their own wood burning. Regarding livestock emissions, residents preferred a large-scale transition to the keeping of fewer animals, whereas farmers preferred the implementation of technical solutions in their stables. Eijrond et al. (2022; [4]) found the same difference in preference in a recent study among farmers and residents in areas with high livestock density in the Netherlands. 

During the dialogue, odour annoyance turned out to be the biggest issue for the residents, even though few reports were made during the project. This is in line with findings from Biesheuvel et al. (2019; [2]) and Eijrond et al. (2022; [4]). They point to a need for more stringent legislation and objective measurement methods. Residents favoured a reduction in livestock intensity to decrease odour annoyance. However, the farmers feared that this would not solve the problem, since the experience of odour is subjective. In this case, the lack of objective data probably hampered the dialogue. After the final session about possible solutions, this project ended. 

At the start of the project, project partners agreed to focus on gaining insight into the local air quality and odour situation, and on having a dialogue involving the farmers and residents. We believe that the methods used in this study could be valuable in similar situations that include multiple (opposing) stakeholders with concerns or questions about air quality. Examples are people that burn wood for domestic heating and those that oppose domestic wood burning, livestock farmers and park rangers, and industrial plants and local residents. In particular the dialogue between stakeholders could foster the sharing of information and facilitate a discussion about possible solutions.

To enable a dialogue between participants based on informed opinions, three things were found to be important: (1) the education of the participants, (2) involvement of independent parties and (3) commitment of the participants.

#### 3.3.1. Education of the Participants

In order to have a conversation about air quality, also based on the insights from the measurements, participants first need to be educated about air quality. They need to know about the drivers of air pollution levels, possible sources of air pollution, measures that impact the emissions from these sources (e.g., COVID-19 restrictions) and the impact of the weather on air quality. In this project, information about air quality was provided by RIVM in presentations (which included time for questions) and interactive sessions in which the participants were actively involved in discussing the results. The Samen Analyseren Tool developed by RIVM was intended to enable participants to analyse their own data, get acquainted with air quality data, and, if possible, formulate their own questions and answers in relation to air quality. However, the tool was used by just a few participants and most of the participants left data analysis and interpretation to the experts from RIVM. The majority of the participants were curious to learn from the experts, and actively participated in the meetings when the measurement results were discussed. In similar citizen sensing projects in which RIVM participated (e.g., the Amsterdam Smart Citizen Lab (ASCL), initiated by Waag Society in 2015), it was found that RIVM experts had intended to take on the role of observers but instead became motivators and trusted sources of information. This was attributed to the fact that a dialogue was established between the scientists and citizen scientists. During the ASCL project, the research team learned a lot about the participants’ needs and wishes, and the participants learned about the scientific process (e.g., gathering data, constructing a data infrastructure). This approach contributed to an increased level of mutual trust, as is stated in the lessons learned by Volten et al. (2018; [15]).

#### 3.3.2. Involvement of Independent Parties

Involving an independent party for the organisation of the project and facilitation of the meetings proved to be valuable, since opposing interests were at stake. RIVM and an independent professional facilitator were viewed as neutral by all of the other stakeholders involved in the project. In the end, participants mostly agreed with the conclusions drawn by RIVM. When asked to formulate conclusions based on the measurement results, farmers and residents sometimes framed their conclusions in a way that served their own interests. For example, farmers specifically focused on the considerable decrease in concentration with increasing distance from the stables, whereas residents concluded that the laying hen stables must be a significant source of pollution since they were the only stables visible in our measurements. The difference between the conclusions is likely due to the nature of citizen sensing projects, in which the participating parties tend to take more of an activist approach than in citizen science projects in general [17,39]. People actively try to bring about a change in policy through measurements, because of the often longstanding distrust of the parties that are held responsible for the problems associated with the aspect being measured [17,40]. The fact that both farmers and neighbours draw conclusions about the measurement results that serve their own interests is to be expected given that they both want different things policy-wise. As an independent party, RIVM focused on the scientific process of measuring and was not involved in any policy choices. This was emphasised several times during the process, and RIVM always carefully chose a position in this regard. This may have contributed to the neutral position the participants attributed to RIVM.

#### 3.3.3. Commitment of the Participants

Also important for the dialogue was the commitment of the participants to the project. From the start of the project, participants were highly committed to performing measurements properly, and also felt responsible for the measurements taken at their premises or in their neighbourhood. They were intrinsically motivated, with a joint interest in obtaining local measurements (rather than models) to characterise their environment. To foster this commitment as much as possible, RIVM encouraged volunteers by making itself available for questions, giving guidance, and showing appreciation for the participants. Additionally, RIVM regularly sent emails to the participants with news, changes in the planning (e.g., due to COVID-19) and announcements. The attendance at the first two plenary meetings (in the second phase), where nearly all participants were present, showed that there was a high level of commitment. However, from the webinars (in the third phase) onwards, the attendance of the residents decreased, with only about 50 to 60 percent of them attending the webinars and meetings. All farmers did attend the meetings throughout the project: since they were just four, we arranged the meeting dates in consultation with them. The decreased attendance might have been partly due to the pandemic. The COVID-19 regulations meant that meetings had to be postponed: two consecutive physical meetings were held 1.5 years apart. We felt that regular physical meetings were important in order to maintain the level of commitment among the participants. People seem to be less willing to attend online meetings within the context of citizen science projects. For example, it has been reported that social factors, such as social bonds and in-person interaction, play a big role in citizen scientists’ motivation to participate [41,42,43]. There is evidence to suggest that these motivating social factors are absent in online citizen science projects, and that people in general are less motivated by online-only interactions [44]. 

## 4. Conclusions

In this study, using low-cost measurement methods, we found that (hourly) PM concentrations were only elevated in the immediate vicinity of layer farms, and not near other type of farms or at residential locations. The NO_2_ levels were higher near road traffic, and the NH_3_ levels were elevated near individual livestock farms and in areas with a high density of livestock. We conclude that low-cost measurement methods for PM, NO_2_ and NH_3_ can be used to characterise local air quality and local farmers and residents can be involved in such efforts. However, neutral guidance is necessary to interpret results from these low-cost measurements, especially concerning the uncertainties of sensors. This might make this method more expensive even though it uses low-cost methods. Furthermore, although participants felt that using the smartphone app for reporting odour annoyance required too much effort, odour annoyance proved to be the most significant concern to residents in this study. Therefore, future research should focus on quantifying odour annoyance with methods that minimise the effort required of the user. 

Our CS approach can help the participants gain valuable insights about the local air quality. For example, the measurements showed that PM concentrations were elevated near layer farms, and information about the participants’ domestic wood burning habits was obtained from the dialogue. These insights can foster a dialogue between different stakeholders, including those who are considered polluters, about ways to improve air quality. Apart from performing measurements together and providing data in a transparent way, we found that education of participants, involvement of independent parties and commitment of the participants are key factors for a constructive dialogue.

Future citizen sensing projects that want to include all stakeholders, including opposing parties, can use our approach, benefit from our experiences and use the lessons learned in this study to improve the design of their sensing projects and maximalise the success rate of such projects. 

## Data Availability

All PM, NO_2_ and NH_3_ measurements were available as open source data on sensors.rivm.nl (accessed on 20 April 2022) during the project. NO2 and NH3 measurements are still available on sensors.rivm.nl (accessed on 20 April 2022). PM data are now accessible through the following API (filter on ‘Boeren en Buren’): https://api-samenmeten.rivm.nl/v1.0 (accessed on 20 April 2022).

## Supplementary Materials

The following supporting information can be downloaded at: https://www.mdpi.com/article/10.3390/s22208053/s1, Table S1: Meetings/interaction with participants of Boeren en Buren Information S1: Comparison of yearly averages of PM2.5 and PM10 at official stations Vredepeel and Horst aan de Maas and the sensors when selecting for humidity ≤95%. Information S2: Uncertainty analysis of collocated SDS011 sensors. Figure S1: PM_10_ hourly average differences between collocated sensor pairs. Red dots show the standard deviation of the absolute difference between collocated sensor pairs, blue dots show the average absolute difference between the sensor pairs. Figure S2: PM_2.5_ hourly average differences between collocated sensor pairs. Red dots show the standard deviation of the absolute difference between collocated sensor pairs, blue dots show the average absolute difference between the sensor pairs. Figure S3: PM_10_ yearly average difference between collocated sensor pairs. Figure S4: PM_2.5_ yearly average difference between collocated sensor pairs.
