# Supplementary material for: Farmers and Local Residents Collaborate: Application of a Participatory Citizen Science Approach to Characterising Air Quality in a Rural Area in The Netherlands
Source: Sensors (Basel). 2022 Oct 21;22(20):8053. doi: 10.3390/s22208053 (PMC9610964; doi:10.3390/s22208053)
Supplement: Supplementary file 1 [file sensors-22-08053-s001.zip › sensors-1922071-supplementary.pdf]

Supplementary Table S1. Meetings/interaction with participants of Boeren en Buren

Meeting	Type	Topics and activities
7 March 2019	Public kick-off meeting	• Presentation of the project set-up by RIVM<br>• Pitches about reasons to participate by RIVM, Municipality of Venray, Province of Limburg, LLTB and GLV
13 May 2019	Meeting with candidate farmer participants	• Explanation of project idea and expectation management by RIVM and LLTB
22 May 2019	Meeting with candidate farmer participants	• Explanation of project idea and expectation management by RIVM and GLV
25 June 2019	Meeting with participants	• Explanation of project design and expectation management by RIVM<br>• Collection of input from participants for the measurement plan<br>• Handing out PM sensors for test measurements in one area
2 October 2019	Meeting with participants	• Individual exercise and plenary discussion about relevant criteria in considerations about livestock farming<br>• Presentation of results test measurements by RIVM + Q&A<br>• Presentation of measurement plan and practicalities by RIVM + Q&A<br>• Handing out PM sensors and materials for measurements of NO2 and NH3
5 December 2019	Panel meeting with 5 residents	• Testing the newly developed smartphone app to report moments of odor annoyance
16 June 2020	Online webinar (due to Covid-19 restrictions)	• Presentation of interim results of the measurements by RIVM + Q&A
31 March 2021	Online webinar (due to Covid-19 restrictions)	• Presentation of end results of the measurements by RIVM + Q&A
7 July 2021	Meeting in small groups of participants (due to Covid-19 restrictions)	• Presentation of general conclusions formulated by RIVM<br>• Interactive ‘play’ with measurement results using transparent pollution rose diagrams to be put on a map of the local situation by participants.<br>• Discussion about new insights to participants<br>• Sharing first ideas about solutions for cleaner air by participants
August 2021	Questionnaire among participants	• Recognition in general conclusions<br>• Solutions for perceived risks and annoyance related to livestock farming
15 September 2021	Meeting with 4 representatives of residents	• Perception of residents about too little recognition for the problem of odor nuisance<br>• Further explanation of measurement results by RIVM

5 October 2021	Meeting with participants	• Addition to general conclusions based on input by participants<br>• Individual exercise and plenary discussion about solution for cleaner air (all sources)<br>• Individual exercise and plenary discussion about effects of different measures for livestock farming on relevant criteria.
20 October 2021	Public end meeting	• Presentation of measurement results by RIVM<br>• Panel discussion about lessons learned by RIVM, Municipality of Venray, Province of Limburg, LLTB and GLV

Supplementary Information S1

Comparison of yearly averages of $PM_{2.5}$ and PM_{10} at official stations Vredepeel and Horst aan de Maas and the sensors when selecting for humidity ≤95%.

The yearly averages of $PM_{2.5}$ and PM_{10} sensor measurements were lower when data with an RH of 95% or under were selected than when the threshold was set to 100% RH and all data were used (Table 1). However, the PM measurements from official monitoring stations at Vredepeel and Horst aan de Maas did not change when the 95% RH filter (Table 1) was applied. This shows that applying the RH filter does not lead to a biased selection of hours for determining the yearly average value.

Supplementary Table S2. Comparison of yearly averages of $PM_{2.5}$ and PM_{10} at two nearby official stations and the sensors when all measurements were included (RH≤100%) and when selected for RH ≤95%. A high RH had no impact on the official measurements. These therefore did not change. Sensors are sensitive to high RH, which in this case could be observed in the decreasing yearly averages when selected for RH ≤95%.

Relative Humidity (RH; %) threshold	$PM_{2.5}$ official measurements ($\mu g/m^3$)	$PM_{2.5}$ sensor measurements ($\mu g/m^3$)	PM_{10} official measurements ($\mu g/m^3$)	PM_{10} sensor measurements ($\mu g/m^3$)
100	10	14	19	30
95	10	11	19	22

Supplementary Information S2

Uncertainty analysis of collocated SDS011 sensors

In our study we used duplicate sensors for each of the 34 measurement locations. As a measure for the uncertainty of the SDS011 PM sensors, we analysed the standard deviation of the calibrated concentration difference between the collocated sensors at all measurement locations (n=34). No specific choices were made regarding the distribution of the sensor boxes. Hours for which the absolute difference between PM_{10} and $PM_{2.5}$ concentration was larger than 100 µg/m^3 were removed from the analyses in order to filter out the clearly erroneous measurements.

Results for hourly averaged concentrations are presented in Figures 1 and 2 in terms of the absolute value of the concentration difference between two collocated sensors. There is variation in the mean absolute difference and the standard deviation between the 34 locations. To quantify the hourly uncertainty of all sensors on all locations in one statistic, a similar analysis was performed using the same data without distinguishing between locations. In this analysis, the 95%CI of the hourly difference between two collocated sensors ranges from -10.7 to 9.9 µg/m^3 for $PM_{2.5}$ and from -17.0 to 18.2 µg/m^3 for PM_{10}.

Note that, due to the sometimes erratic/chaotic behaviour of the sensors, the standard deviation of the hourly differences between the collocated sensors usually lies between the 75% and 95% percentiles of the data (Figure 1&2). This indicates a deviation of the distribution of the hourly concentration differences from the normal distribution.

From the selected hourly data, yearly averaged concentrations were calculated for each sensor. Subsequently the difference between the two collocated sensors was calculated for each location. The yearly averaged differences are presented in Figures 3 and 4. The 95%CI of the yearly averaged difference between two collocated sensors, calculated over the 34 locations and assuming a normal distribution, ranges from -4.4 to 4.4 µg/m^3 for $PM_{2.5}$ and from -6.8 to 6.8 µg/m^3 for PM_{10}.

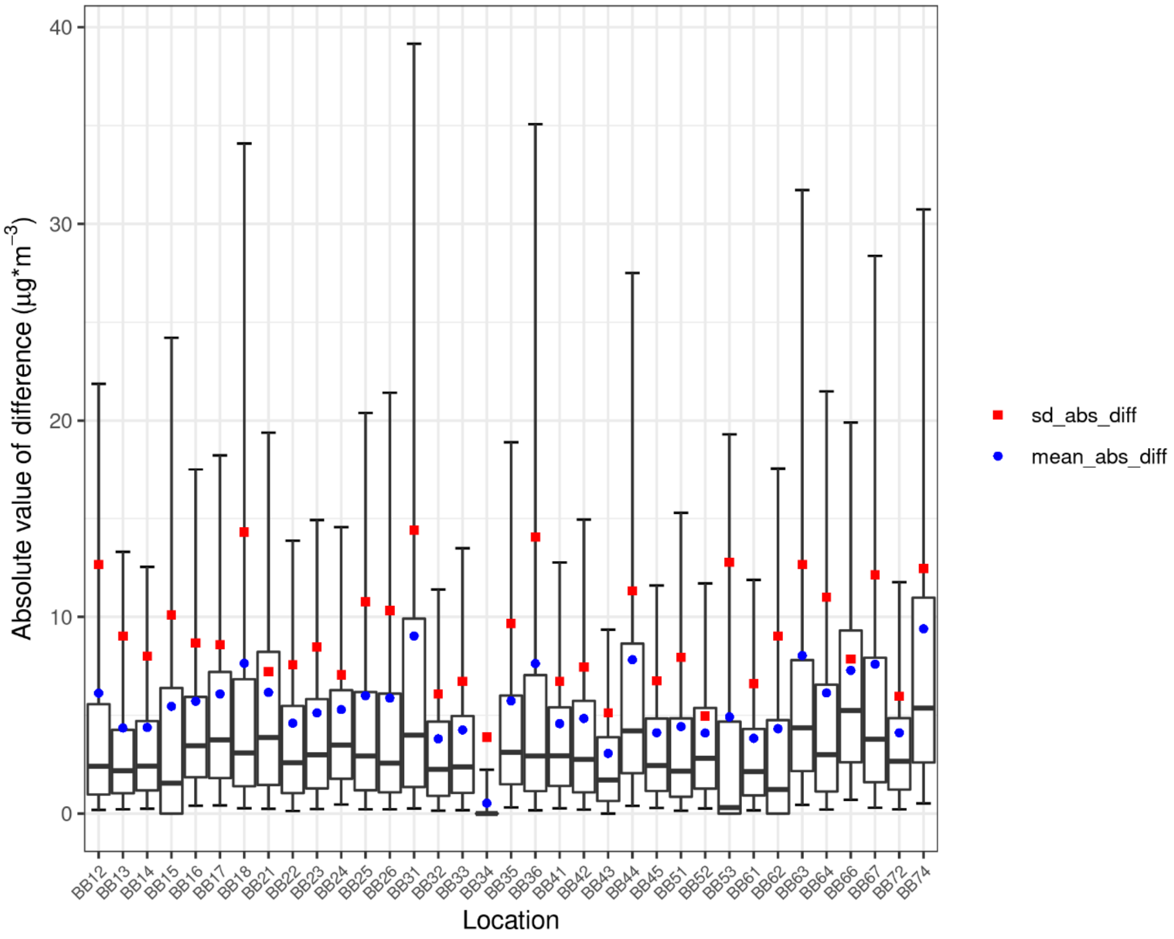

Supplementary Figure S1. PM_{10} hourly average differences between collocated sensor pairs. Red dots show the standard deviation of the absolute difference between collocated sensor pairs, blue dots show the average absolute difference between the sensor pairs.

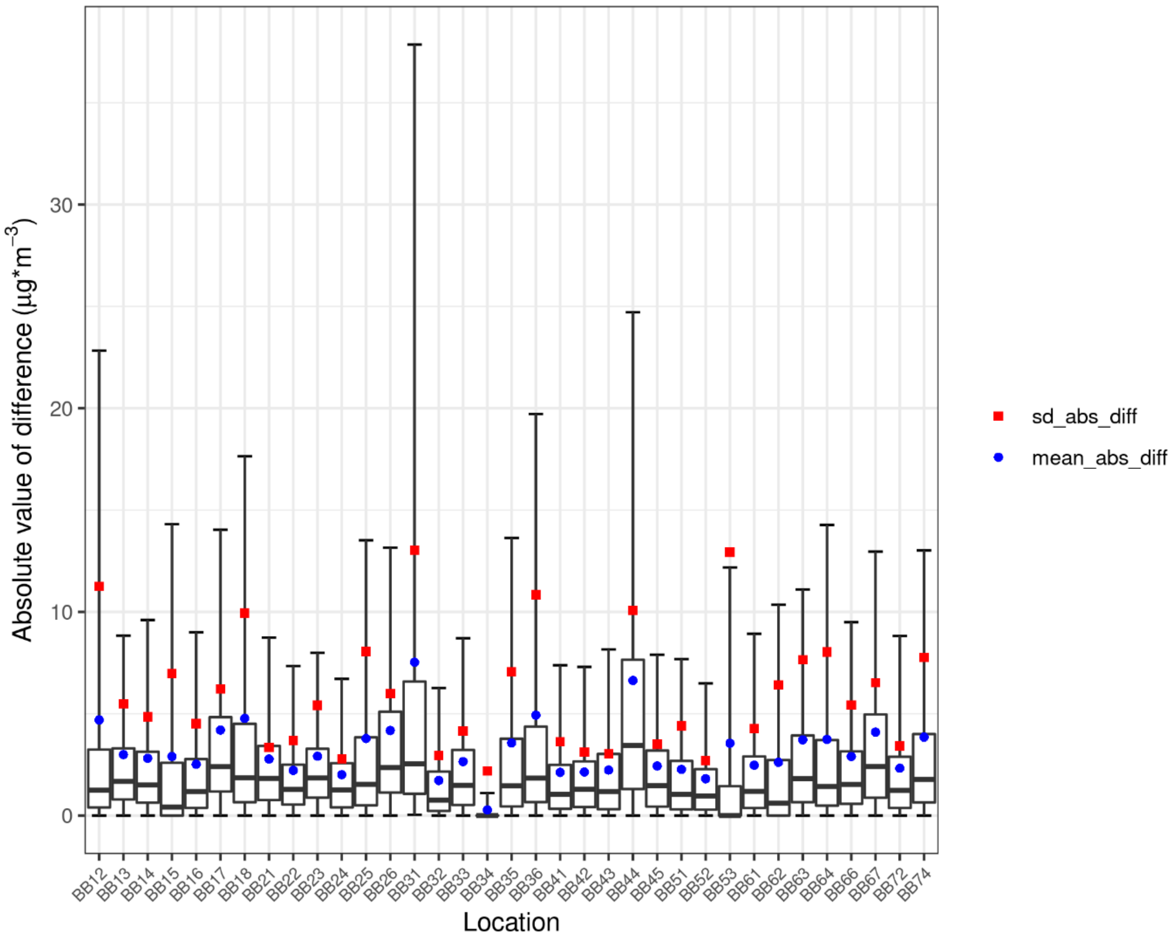

Supplementary Figure S2. $PM_{2.5}$ hourly average differences between collocated sensor pairs. Red dots show the standard deviation of the absolute difference between collocated sensor pairs, blue dots show the average absolute difference between the sensor pairs.

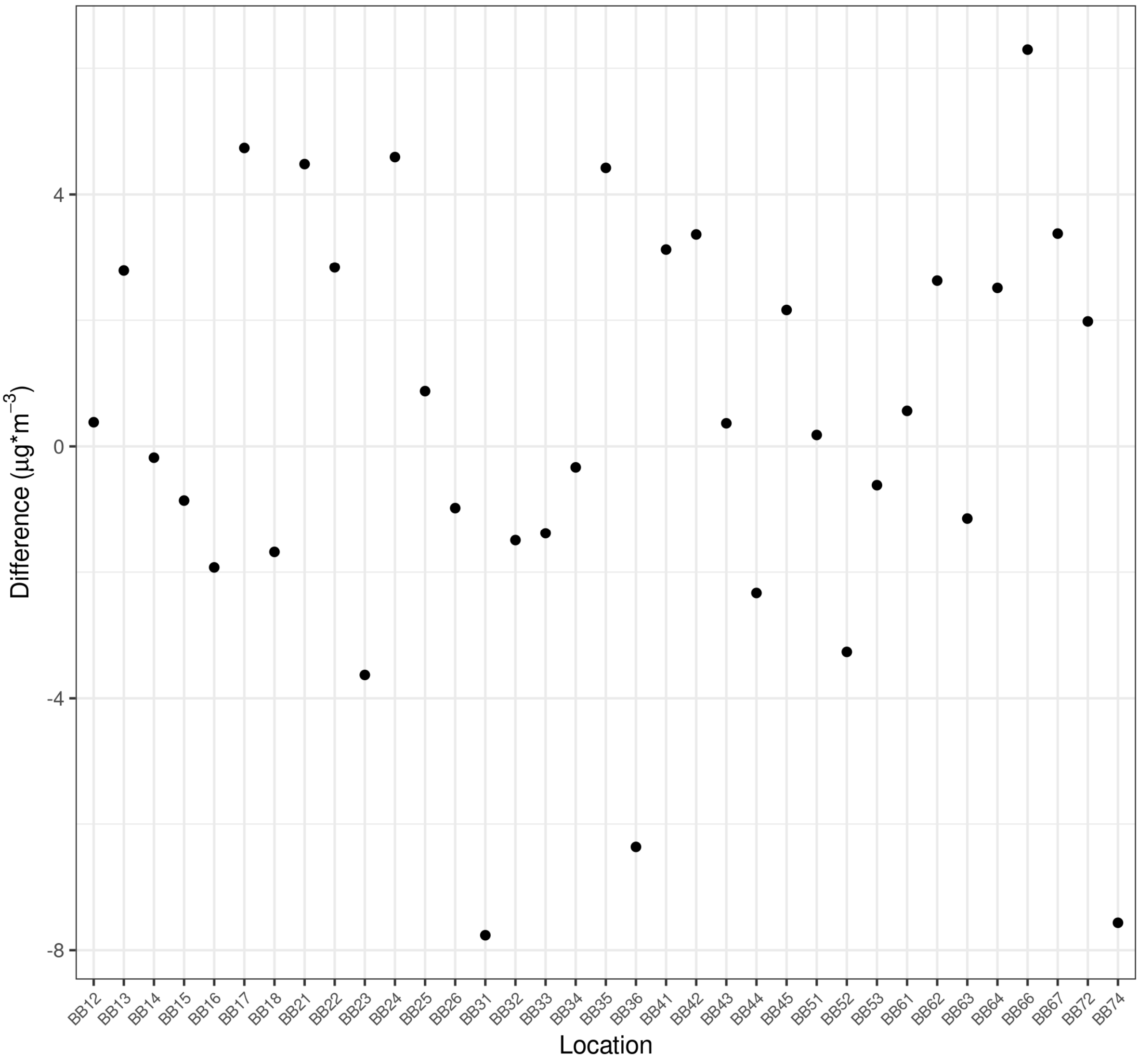

Supplementary Figure S3. PM_{10} yearly average difference between collocated sensor pairs.

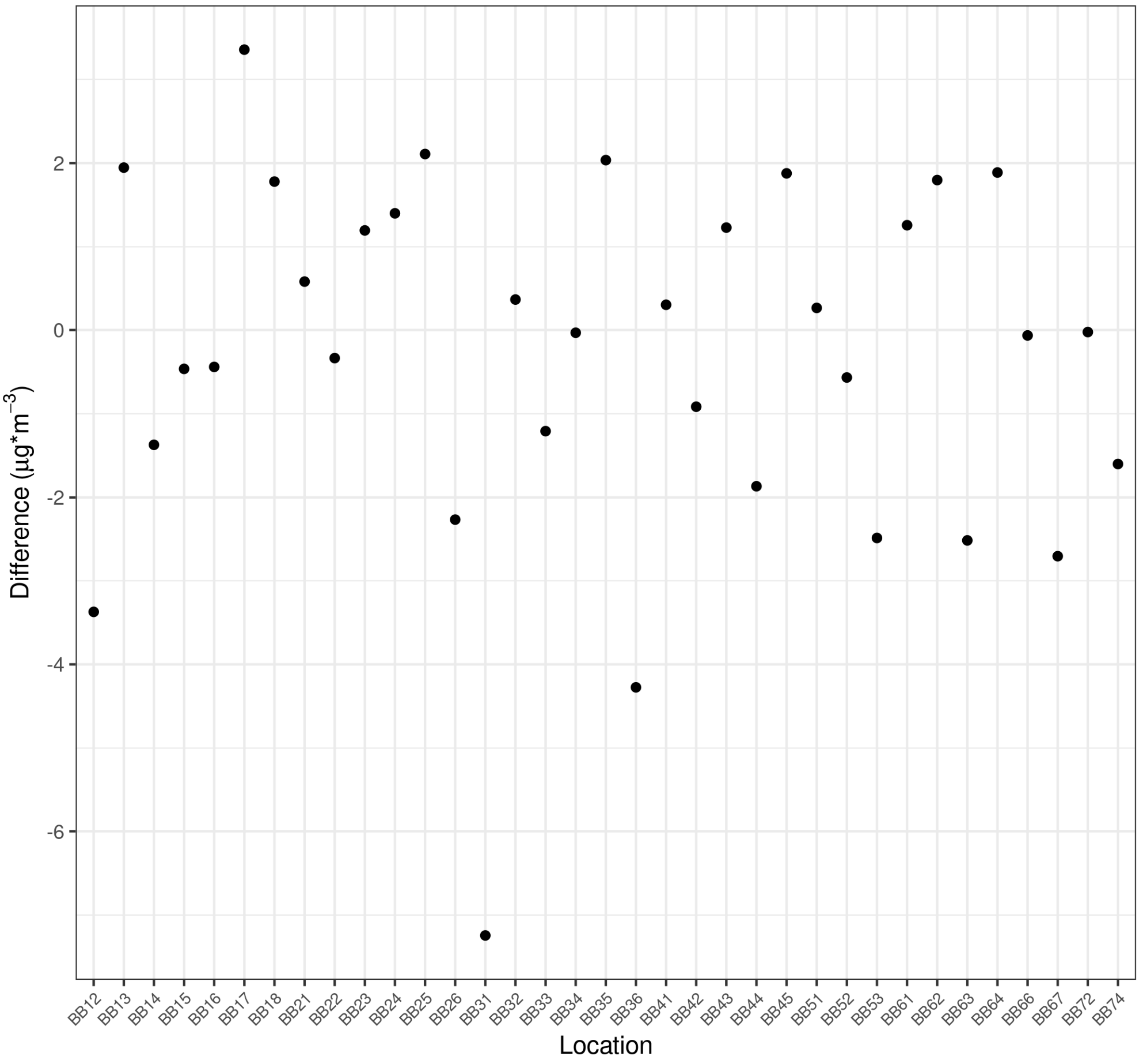

Supplementary Figure S4. $PM_{2.5}$ yearly average difference between collocated sensor pairs.
